# SpliPath enhances disease gene discovery in case-control analyses of rare splice-altering genetic variants

**DOI:** 10.1016/j.crmeth.2025.101176

**Published:** 2025-09-17

**Authors:** Yan Wang, Charlotte van Dijk, Ilia Timpanaro, Paul Hop, Brendan Kenna, Maarten Kooyman, Eleonora Aronica, R. Jeroen Pasterkamp, Leonard H. van den Berg, Johnathan Cooper-Knock, Project MinE ALS Sequencing Consortium, NYGC ALS Consortium, Jan H. Veldink, Kevin Kenna

**Affiliations:** 1 Department of Translational Neuroscience, UMC Utrecht Brain Center, University Medical Center Utrecht, 3584 CG Utrecht, the Netherlands; 2 Department of Neurology, UMC Utrecht Brain Center, University Medical Center Utrecht, 3584 CX Utrecht, the Netherlands; 3 Department of (Neuro)Pathology, Amsterdam UMC, University of Amsterdam, 1105 AZ Amsterdam, the Netherlands; 4 Sheffield Institute for Translational Neuroscience (SITraN), University of Sheffield, Sheffield S10 2HQ, UK

**Keywords:** RNA splicing, splice-altering variant, collapsed rare variant splicing quantitative trait locus, intronic mutation, gene burden test, rare variant association test, missing heritability, rare disease, ALS

## Abstract

We developed SpliPath as a generalizable framework to discover disease associations mediated by rare variants that induce experimentally supported mRNA splicing defects. Our approach integrates components of burden tests (BTs), traditional splicing quantitative trait locus (sQTL) analyses, and sequence-to-function AI models (SpliceAI and Pangolin). Central to the workings of SpliPath is our concept of collapsed rare variant splicing QTL (crsQTL). crsQTL groups rare variants that are predicted to alter splicing in the same way, specifically by linking them to shared splice junctions observed in independent (unpaired) RNA sequencing (RNA-seq) datasets. We demonstrate the utility of SpliPath through applications in amyotrophic lateral sclerosis (ALS). Through this, we showcase scenarios where SpliPath detects genetic associations that cannot be recovered by more simplistic combinations of BT and SpliceAI. We also nominate crsQTL for splice defects detected in large-scale analyses of ALS patient tissue.

## Highlights

• SpliPath is a tool to discover rare splicing defects associated with human disease
• Combines rare variant burden testing, RNA-seq analyses, and sequence-to-function AI models
• Detects disease gene associations missed by conventional gene burden testing
• Identifies disease-associated collapsed rare variant splicing QTL (crsQTL) in ALS

## Motivation

Bioinformatic analyses of RNA sequencing (RNA-seq) data can reveal rare splicing events in disease models and patient tissues, but it can be challenging to establish which events do and do not contribute to disease processes. Genetic analyses can be used to test for direct associations between disease risk and rare splice-altering DNA variants. For the latter, burden tests can aggregate statistical evidence across rare variants while sequence-to-function models (e.g., SpliceAI and Pangolin) can predict which rare variants disrupt splicing. However, here we demonstrate that simplistic combination of burden testing with SpliceAI can fail to recover important associations in real-world data. We therefore developed SpliPath, a tool that provides missing functionalities to interpret rare splicing events detected in RNA-seq by revealing rare variant disease associations in independent (unpaired) genetic datasets.

## Graphical abstract

**Figure:**
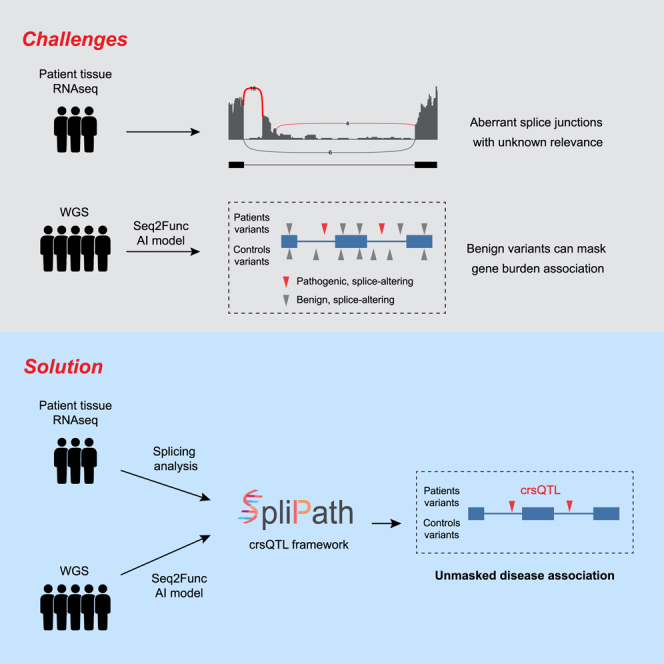

## Teaser

Wang et al. introduce SpliPath, a method to discover gene splicing defects associating with human disease using rare variant analyses. SpliPath combines independent genetic and transcriptomic datasets with sequence-to-function AI models, providing a tool to enhance discovery in rare variant disorders such as ALS.

## Introduction

Mapping missing heritability can be challenging for disorders that typically lack clear Mendelian inheritance patterns but are nonetheless primarily driven by rare genetic variants. Amyotrophic lateral sclerosis (ALS) is one such disorder, with an estimated heritability of ∼60% from twin studies, yet only ∼10% of patients have a known family history.^1^ Despite this, ALS genome-wide association studies (GWASs) have revealed notably fewer hits than similarly sized GWASs of other CNS disorders.^2^^,^^3^^,^^4^ Heritability partitioning analyses have revealed striking enrichment for genetic risk in rarer minor-allele frequency bins.^4^^,^^5^ Segregation-based analyses of the 10% of patients with a familial history have revealed multiple high-effect-size disease genes, but these explain only a minority of ALS cases. We and others have demonstrated that rare variant collapsing or burden tests (BTs) provide an effective alternative to drive novel disease gene discovery in unbiased analyses of large case-control cohorts.^6^^,^^7^^,^^8^ However, BTs have important limitations that impact their capacity to detect genes with differing genetic architectures, and new methods are required to overcome this challenge and further advance genetic discoveries in rare variant disorders.

The key challenge of BTs lies in the selection of “qualifying variants.”^9^ The inclusion of benign variants will obscure association signals from *bona fide* disease variants and cause a loss of sensitivity for disease gene discovery. Conversely, excessive stringency in selecting qualifying variants will attenuate power for disease gene discovery by excluding *bona fide* disease variants.^9^ Ideally, if variant effects could be predicted with high accuracy, then grouping variants by shared functional consequences would enhance gene discovery. Sequence-to-function AI models such as SpliceAI^10^ or Pangolin^11^ have provided a remarkable advance in our capacity to predict the functional consequences of genetic variants on mRNA splice junction usage. For genes where any splice junction disruption is pathogenic, SpliceAI alone might suffice as a filter for well-powered BT analyses. However, when only a subset of splice junction disruptions is pathogenic, then the inclusion of non-pathogenic splice-altering variants in BT analyses can again continue to mask disease associations. This can be especially relevant for genes where only gain-of-function or more specific partial loss-of-function effects contribute to disease.^12^

We hypothesize that, if rare variants detected in patients can be mapped to splice alterations detected in disease-relevant transcriptomics datasets, then functionally equivalent variants can be clustered based on a shared splicing phenotype. These clusters, which we refer to as collapsed rare variant splicing quantitative trait loci (crsQTLs), could then be tested for disease association. The use of independent reference transcriptomics data in this process would allow variant clustering to be targeted to splice junction consequences that have independent experimental support. This approach has obvious parallels to traditional splicing QTL (sQTL) analyses used in GWASs^13^ and could help in addressing the lack of methods to extend sQTL analyses from common variants to rare variants. Therefore, we developed SpliPath, a computational framework that uses reference transcriptomics datasets to functionally cluster splice-altering variants from independent whole genome sequencing (WGS) datasets into crsQTLs. In this study, we applied SpliPath to two ALS-related WGS datasets and two distinct categories of disease-relevant transcriptomic data. Through these analyses, we explored the discovery of ALS crsQTLs using reference data from large-scale RNA sequencing (RNA-seq) of ALS patient tissues and reference data from laboratory models, and we contrasted our approach with the results achieved using simpler BT analyses with standard SpliceAI filters.

## Results

### Overview of the SpliPath framework

**Figure 1:**
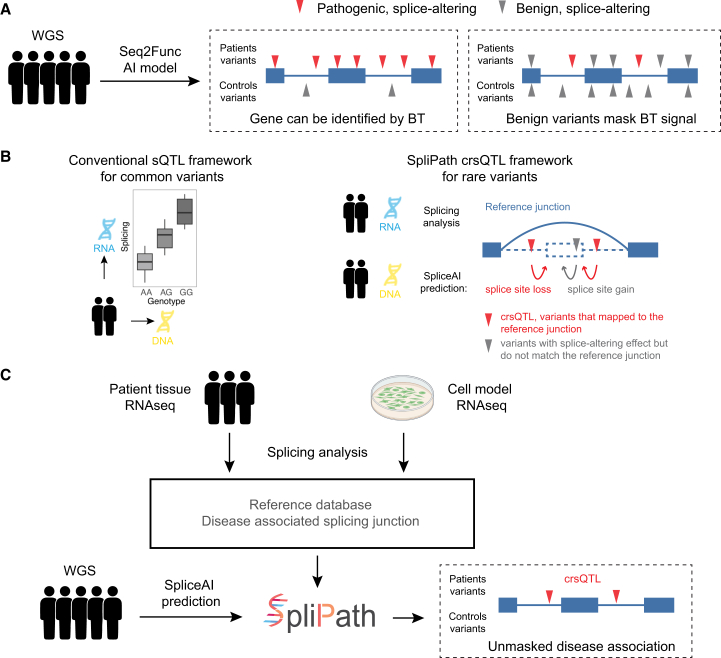
SpliPath framework for identifying rare variant crsQTL (A) Burden tests (BTs) are an effective approach for identifying disease-associated genes but rely on correctly selecting “qualifying variants.” The inclusion of benign or wrongly predicted splice-altering variants can mask association signals from pathogenic mutations, reducing sensitivity for disease gene discovery. (B) Traditional sQTL studies identify statistical associations between common DNA variants and splicing phenotypes using reference transcriptomics datasets with paired genetic information. This approach is not feasible for rare variants due to limited sample sizes. SpliPath overcomes this challenge by matching rare variants with experimentally observed splicing junctions that align with SpliceAI-predicted splice site gains or losses. (Blue arc, exon-skipping splice junction observed in the reference RNA-seq datasets; dashed lines, skipped exon; curved arrows, location of a splice site that is lost/gained due to variant.). (C) The application of SpliPath for the discovery of rare splice-altering variant associations involves 3 steps: (1) quality control and annotation of splice junctions detected in reference transcriptomics datasets, (2) annotation of case-control WGS datasets using sequence-to-function AI prediction tools, and (3) crsQTL clustering to discover novel associations with disease traits.

SpliPath is designed to identify and functionally cluster rare genetic variants that collectively associate with a disease trait because of a common influence on a shared mRNA splicing defect. We refer to these collective variant sets as crsQTLs, and the core hypothesis of SpliPath is that crsQTL analyses provide a more powerful alternative to traditional BT analyses in scenarios where only a subset of predicted splice- altering variants is truly pathogenic (Figure 1). The first step of this process involves the generation and quality control of a reference database that summarizes positional information and statistical analyses for mRNA splice junctions expressed in disease-relevant patient tissues or laboratory models. The second step involves predicting splice-altering effects for rare variants in a case-control genetic dataset. This prediction step uses sequence-to-function AI models, SpliceAI or the related model Pangolin, to predict whether and how genetic variants impact the probabilities of local splice site usage. More specifically, these predictions include estimated probabilities for new donor site gain, donor site loss, new acceptor site gain, and acceptor site loss events. The final step then involves the clustering of variants that are linked to the same candidate splice junctions for crsQTL association testing. This framework has obvious parallels with traditional sQTL analyses used for common variants in GWASs,^13^ except that sequence-to-function AI models are used to link rare variants sets with the reference transcriptomics dataset. SpliPath supports 2 tiers of matching between SpliceAI (or Pangolin) predictions and reference splice junctions. These include “full match” events, where a reference splice junction matches fully with a set of SpliceAI predictions, and “partial match” events, which are described in detail in the STAR Methods and Figure S1. The functionalities of SpliPath have been implemented in an R package that includes a data browser for interactive analysis and visualizations as well as accompanying tutorials (https://github.com/KennaLab/SpliPath; Figure S2).

### Applying SpliPath for crsQTL discovery from paired WGS–RNA-seq profiling of patient tissues

**Figure 2:**
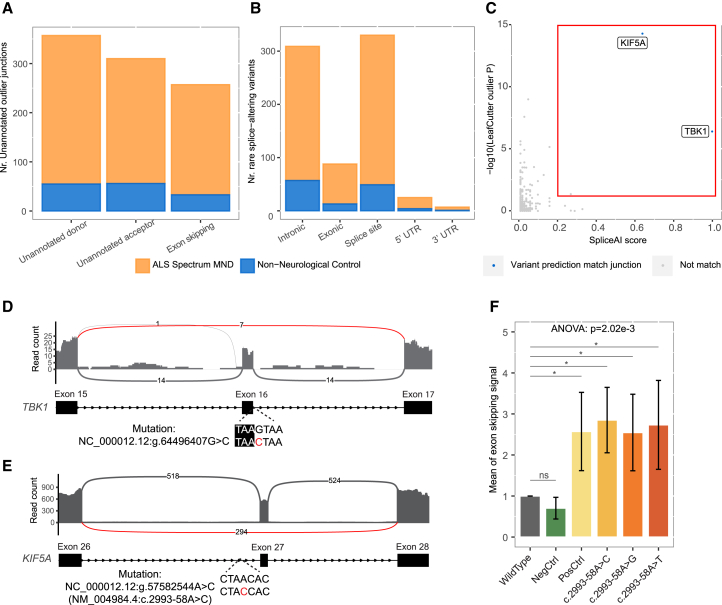
Identifying and validating crsQTLs linked to known pathogenic splicing events detected in patient tissues (A) Distribution of observed splice junctions in ALS patients and non-neurological disease controls across different junction categories, identified by crsQTL analysis of a paired NYGC WGS-RNA-seq dataset. (B) Distribution of the locations of the rare variants involved in the crsQTL. (C) Scatterplot depicting SpliceAI scores (*x* axis) and LeafCutterMD *p* values (*y* axis) for tentative pairings of rare variants and splice junctions (motor cortex) that occur within the same subgenic windows (STAR Methods) of known ALS genes (Figure S6A) in the same sample. Junction-variant pairings that fulfill SpliPath matching criteria are colored blue, whereas junction-variant pairings that do not fulfill SpliPath matching criteria are colored gray. Two junction-variant pairings (*TBK1* and *KIF5A*) were classified as crsQTLs having fulfilled SpliceAI matching criteria as well as the prefiltering thresholds of LeafCutterMD *p* <0.05 and a SpliceAI score ≥0.2. (D) RNA-seq coverage for the expression of *TBK1* and crsQTL mapped to the observed exon 16-skipping event. (E) RNA-seq coverage for the expression of *KIF5A* and crsQTL mapped to the observed exon 27-skipping event. (D and E) The arcs and numbers on them show the number of reads mapped to splicing junctions, and the red arcs show the reads mapped to unannotated splicing junctions representing skipping of exons. (F) Bar plot depicting results from the *KIF5A-exon27* minigene reporter assay. No differences in exon skipping signals were observed between cells expressing the wild-type *KIF5A* sequence and a negative control variant (c.2993-12T>C, from the gnomAD non-neurological disease group). All variants at the suspected *KIF5A-exon27* branchpoint exhibited significant elevations in exon skipping signals, as did a positive control variant (c.3020+3A>G) disrupting the 5′ consensus splice site (∗*p*.adj < 0.05, t test, false discovery rate [FDR] adjusted; error bars represent mean ± standard deviation).

GTEx and other consortia have generated large catalogs of common variant sQTLs from large-scale bulk RNA-seq of human tissues and paired genetic data. To explore a parallel application for rare variant csQTLs, we next used SpliPath to build a reference database of candidate ALS splice junctions using paired RNA-seq and WGS data from the New York Genome Center (NYGC) cohort (294 cases, 76 controls, RNA-seq of 1–4 brain and spinal cord tissues per donor). To achieve this, we first established a catalog of rare splice junctions represented in the RNA-seq data. For this, we used LeafCutterMD^14^ to identify junctions that exhibited a nominally significant *p* value for outlying expression and removed events represented within external reference datasets of annotated splice junctions (STAR Methods). We observed no significant difference in the global frequency of prioritized splice junctions in patients vs. controls for any of the 4 tissues (frontal cortex, *p* = 0.81; motor cortex, *p* = 0.24; cervical cord, *p* = 0.08; lumbar cord, *p* = 0.47; t test; Figure S3). We then applied SpliPath in a paired data analysis mode to recover 755 junctions from patient tissues where paired WGS data for the same samples supported the presence of matching *cis*-acting splice-altering variants (Figures 2A and 2B; Table S1). Results from corresponding analyses of control samples are also provided in Table S1).

Amongst these 755 crsQTL junctions, we noticed 2 events in known ALS genes that were recognizable as likely pathogenic (Figure 2C). The first variant disrupted a consensus splice site in the *TBK1* gene (NC_000012.12:g.64496407G>C, SpliceAI score = 1.0, *n* = 1 patient; Figure 2D). The second was a rare variant located 58 bp away from the intron-exon boundary of exon 27 in the *KIF5A* gene (NC_000012.12:g.57582544A>C/c.2993-58A>C, SpliceAI score = 0.64, *n* = 1 patient; Figure 2E). Selective skipping of exon 27 in *KIF5A* has previously been implicated in ALS,^8^ but interestingly, the variant in question occurred deeper in the intronic sequence than what is captured when using precomputed SpliceAI scores or other consensus junction-focused tools. Using a branch point prediction tool (BPP),^15^ we predicted that the *KIF5A* crsQTL corresponded to a branchpoint disruption. Lookup of this position in an independent ALS genetic dataset (Project MinE) revealed 2 additional carriers of probable branchpoint-disrupting variants (c.2993-58A>C, *n* = 1 patient; c.2993-58A>G, *n* = 1 patient). To validate that all possible SNVs (including the A>C and A>G found in patients and another A>T) at this predicted branchpoint were indeed associated with exon 27 skipping, we designed a minigene reporter assay based on a construct used in a recent massively parallel splice reporter assay^16^ (Figure S4). This assay confirmed the expected increases in exon skipping for all branchpoint variants (Figure 2F) as well as expected results for an additional positive control variant (c.3020+3A>G, intron-exon boundary variants reported in previous work^8^) and negative control variant (c.2993-12T>C from the gnomAD non-neurological disease group, allele count = 5; Table S2). Collectively, our observations for *TBK1* and *KIF5A* confirm that SpliPath provides a convenient framework to recover disease-relevant crsQTL from a paired WGS-RNA-seq dataset.

### crsQTL analyses of WGS data expand disease gene discovery in case-control association studies

**Figure 3:**
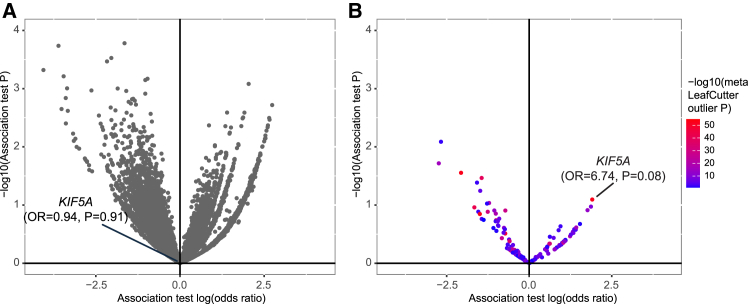
SpliPath-informed crsQTL association tests extend disease gene discovery in genome-wide case-control analyses (A) Volcano plot depicting log odds ratios (ORs) and *p* values from gene BT analyses of Project MinE data with prefiltering for the 142,058 variants assigned precomputed SpliceAI scores ≥0.2. (B) Volcano plot depicting log ORs and *p* values from crsQTL-informed association testing of Project MinE. These analyses include 479 SNVs linked to a set of 755 reference splicing junctions derived from ALS patient tissues. The dots are colored based on LeafCutterMD *p* values observed for the corresponding splice junctions in patient RNA-seq. These *p* values are meta *p* values, combined from LeafCutterMD *p* values of splice junctions across multiple tissues. All association analyses are restricted to genes/crsQTLs that have at least 3 observations in Project MinE.

Unlike conventional sQTL analyses that require paired RNA-seq and WGS from the same individuals, SpliPath enables the discovery of splice-altering variants (crsQTL) in large WGS cohorts that can induce disease-associated splice junctions independently detected in any reference RNA-seq dataset. These crsQTL in large WGS cohorts can then be tested for disease associations using BTs. We next applied SpliPath to the larger Project MinE WGS dataset (6,625 ALS patients and 2,472 controls), using the 755 splice junctions identified from the NYGC dataset as our reference, and compared its power to a SpliceAI-informed BT for disease gene discovery. Our analysis identified 479 SNVs (carrier frequency <0.1%) in Project MinE WGS, which can be assigned to one of these reference junctions from NYGC RNA-seq (Table S3). In contrast, a total of 142,058 rare SNVs achieved precomputed SpliceAI scores ≥0.2 in the same dataset. We then used Firth logistic regression to test each crsQTL for association with ALS risk (Figure 3; Table S4). For comparison, we also performed gene burden analyses that were restricted to all variants with precomputed SpliceAI scores ≥0.2 (Figure 3; Table S4). The *KIF5A* exon 27 skipping crsQTL is readily discoverable as a lead candidate for further validation in crsQTL-informed association testing. In total, this crsQTL aggregates 6 independent variants that are observed within 8 patients and 0 controls (Table S5). These data are in line with a high odds ratio known to be associated with exon 27 skipping.^8^ Conversely, the *KIF5A* association is in no way observable when simple SpliceAI based gene burden testing is applied (odds ratio [OR] = 0.94, *p* = 0.91; Figure 3).

To further validate this observation, we also performed targeted analyses of the *KIF5A* gene in a larger whole-exome sequencing (WXS) cohort (Project MinE, *n* = 12,905 patients and 69,718 controls, unpublished data, P.H., J.H.V., K.K.). In this cohort, the *KIF5A* exon 27-skipping crsQTL collapsed 9 independent variants, observed within 10 patients and 2 controls (OR = 20.66, *p* = 8.18 × 10^−7^’ Tables 1 and S5), exceeding standard exome-wide significance criteria. In contrast, the SpliceAI-informed burden test did not reach significance (OR = 2.36, *p* = 1.02 × 10^−2^) because it aggregated 26 predicted splice-altering variants found in 16 patients and 30 controls, many of which were not linked to disease risk (Table S5). The crsQTL-informed approach outperformed the SpliceAI-informed approach by selectively grouping variants that drive pathogenic splicing events, avoiding dilution from irrelevant variants. We also repeated these comparisons where other splice prediction models were used in place of the default SpliceAI model, including SpliceAI-D4999 (STAR Methods), Pangolin,^11^ and SpliceTransformer.^17^ Notably, both Pangolin and SpliceTransformer used brain-specific datasets during model training, which might be expected to enhance performance for a disorder like ALS. However, in all cases, notable association signals for the *KIF5A* gene were only achieved when using the SpliPath crsQTL framework (Table 1).

**Table 1:** Extended comparison of methods to discover association between rare splice variants and disease risk in a large ALS WXS dataset

Variants selection methods	*KIF5A*			*NEK1*		
	No. of variants|no. of case carriers|no. of control carriers^a^	OR	*p* Value	No. of variants|no. of case carriers|no. of control carriers^a^	OR	*p* Value
SpliceAI-D50	26|16|30	2.36	1.02 × 10^−2^	38|41|95	2.60	2.87 × 10^−6^
SpliceAI-D500	30|17|33	2.28	1.09 × 10^−2^	51|49|112	2.58	4.66 × 10^−7^
SpliceAI-D4999	30|17|33	2.28	1.09 × 10^−2^	51|49|112	2.58	4.66 × 10^−7^
SpliceTransfomer	13|6|19	1.28	0.60	14|13|18	4.24	2.24 × 10^−4^
Pangolin	23|9|27	1.32	0.49	34|46|89	3.02	3.12 × 10^−8^
SpliPath-Pangolin	6|6|2	12.45	4.68 × 10^−4^	–	–	–
SpliPath-SpliceAI	9|10|2	20.66	8.18 × 10^−7^	–	–	–

a The association tests were performed on the Project MinE WXS cohort comprising 12,905 ALS patients and 69,718 controls.

It is important to note that the superior performance of SpliPath in recovering genes such as *KIF5A* is specific for scenarios where the ratio of pathogenic splice-altering variants to non-pathogenic splice-altering variants is comparatively low (Figure 1A). To illustrate this point, we therefore also repeated our targeted analyses for *NEK1*, a gene where it is anticipated that most splice-altering variants throughout the gene are associated with increased disease risk.^7^ Here, significant associations with ALS risk were observed using SpliceAI- or Pangolin-informed BTs. In this case, SpliPath failed to provide a testable cluster of crsQTL, since no *NEK1* splice junction passed QC in the reference database (Table 1). Collectively, these results demonstrate that SpliPath and BT are complementary and argue for coordinated use of both approaches in the discovery of novel rare variant association signals at splice-altering variants.

**Figure 4:**
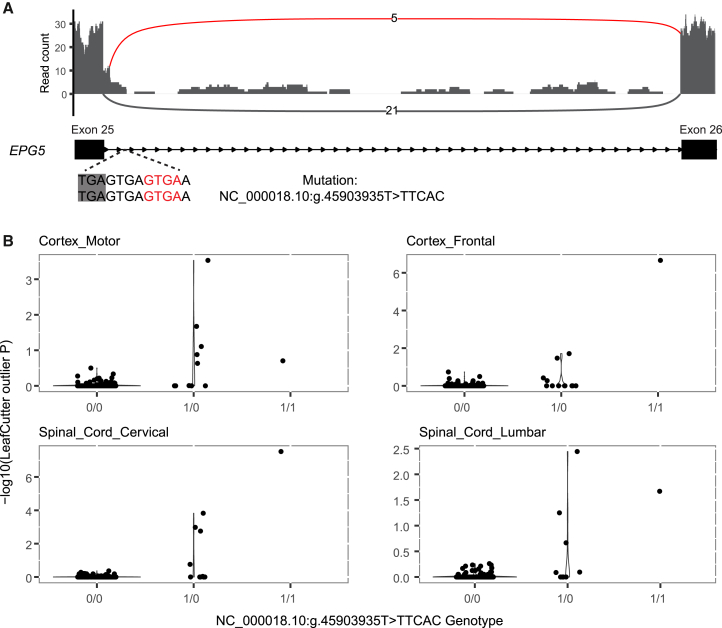
The *EPG5* crsQTL junction correlates with genotype dosage in RNA-seq of ALS patient tissues (A) Coverage and splice junction usage within RNA-seq of frontal cortex tissue from a carrier homozygous for NC_000018.10:g.45903935T>TTCAC insertion. Red arcs depict reads supporting an elongation of exon 25. *EPG5* locates on the antisense strand; its orientation has been mirrored so that the gene is displayed in the 5′ to 3′ direction. (B) LeafCutterMD *p* values for expression of the *EPG5* exon 25 extension junction across different genotypes of the NC_000018.10:g.45903935T>TTCAC insertion.

Within the Project MinE WGS dataset, we observed the *KIF5A* crsQTL SNVs in a total of 8 patients and 0 controls. The sample size was not sufficient for this to yield a significant crsQTL association, and a significant *p* value was also not achieved by any other known or novel ALS gene. We did, however, note 7 other genes where the variants linked to NYGC crsQTLs were both absent from controls and present in ≥5 patients (Table 2). These genes included *EPG5*, which is notable, given that *EPG5*^−/−^ mice have previously been reported to exhibit key ALS characteristics, including motor neuron loss, TDP-43 aggregation in motor neurons, and muscle atrophy.^18^^,^^19^^,^^20^ Our analyses nominated *EPG5* under a recessive disease model, with 6 patients homozygous for an NC_000018.10:g.45903935T>TTCAC variant. The variant causes elongation of exon 25 (Figure 4) and is predicted to introduce an insertion of 11 amino acids in protein. The validity of the association between this variant and altered splicing was further supported by a significant correlation between junction usage and variant dosage in the NYGC dataset (356 non-carriers, 13 heterozygotes, 1 homozygote; *p* < 2 × 10^−16^ in all 4 tissues; Figure 4). However, in the larger Project MinE WXS cohort, the variant showed only a marginal association with ALS risk and a markedly diminished effect size (OR = 2.54, *p* = 0.039); a definitive confirmation or exclusion of the association will thus require a large independent replication cohort.

**Table 2:** Top candidate csQTLs from SpliPath annotation of the Project MinE WGS cohort

Gene	Prior evidence for ALS relevance	Event	Junction	Analysis	No. of variants	No. of case carriers	No. of control carriers	*p* Value
*KIF5A*	Nicolas et al.^8^	exon skipping	12:57581952:57583100:+	allelic, SNV only	6	8	0	0.0804
*KLHDC9*	no	exon skipping	1:161099062:161100060:+	allelic, SNV + indel	5	8	0	0.0713
*KLHDC9*	no	exon skipping	1:161099062:161100060:+	allelic, SNV only	4	7	0	0.1225
*EPG5*	Zhao et al.^18^ Zhao et al.^20^	unannotated donor site	18:45901167:45903939:–	recessive, SNV + indel	1	6	0	0.2259
*FAM102B*	no	unannotated acceptor site	1:108629657:108635430:+	recessive, SNV + indel	1	6	0	0.1773
*SLC25A46*	no	unannotated donor site	5:110748678:110755464:+	allelic, SNV + indel	2	5	0	0.2883
*NPRL3*	no	unannotated donor site	16:88890:89764:-	allelic, SNV + indel	2	5	0	0.2428
*EDIL3*	no	exon skipping	5:84180521:84384307:-	allelic, SNV + indel	1	5	0	0.4871
*NINJ1*	no	unannotated donor site	9:93125062:93125656:-	allelic, SNV + indel	1	5	0	0.1065
NINJ1	–	unannotated acceptor site	9:93125749:93126409:-	allelic, SNV + indel	1	5	0	0.1065

Top events include crsQTLs observed in 5 or more patients but 0 controls. A complete list of crsQTLs with case-control frequencies is available in Table S4. Indel, insertion or deletion.

### SpliPath detects verifiable crsQTLs in WGS using reference splicing data from cell models

**Figure 5:**
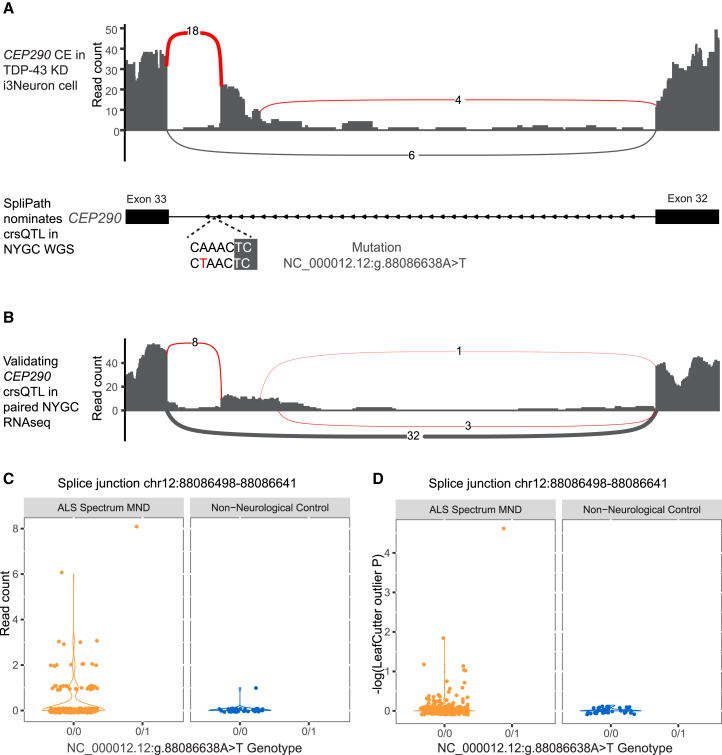
Discovery and validation of a crsQTL for a CE retention event in *CEP290* (A) RNA-seq coverage for the expression of a *CEP290* CE-relevant junction within a TDP-43 KD i^3^Neuron cell model. SpliPath nominated a crsQTL (NC_000012.12:g.88086638A>T) for this event in WGS of an independent ALS patient from the NYGC cohort. (B) Validation that the patient carrying the crsQTL indeed exhibits expression of the expected CE retention in paired RNA-seq (motor cortex; see also Figure S4). (C) The read coverage per sample within the NYGC cohort at the *CEP290* CE junction (motor cortex). (D) Per-sample LeafCutterMD *p* values to test the statistical significance of outlier splicing within the NYGC cohort at the *CEP290* CE (motor cortex).

Above, we used splice junctions derived from RNA-seq of ALS patient tissues to map crsQTL for WGS data first from a paired study cohort (NYGC) and then from an unpaired cohort (Project MinE). Since SpliPath does not formally require paired genetics data for the selected source of splice junctions, it is, in theory, feasible to go beyond RNA-seq of human cohorts and build crsQTL for splice junctions detected in artificial laboratory models. This is an important contrast versus traditional common variant sQTL mapping paradigms. To explore this, we curated a set of cryptic exon (CE) retention events from RNA-seq of a well-known cell culture model of TDP-43 dysfunction.^21^ This model recapitulates a key neuropathological hallmark of ALS, and generated CEs include key players in ALS pathobiology (CEs in *STMN2* and *UNC13A*).^21^^,^^22^ SpliPath nominated 53 variants across the NYGC and Project MinE cohorts that were tentatively linked to one of these CEs (Table S6). Among these, we noted an NC_000012.12:g.88086638A>T variant carried by a patient from the NYGC cohort, where paired postmortem RNA-seq revealed a statistically significant increase in the exact *CEP290* CE predicted using SpliPath analyses (*p* = 2.18 × 10^−5^, LeafCutterMD outlier analysis; Figures 5 and S5). However, our analyses also revealed important caveats of this approach. For example, neither SpliceAI nor Pangolin was capable of predicting the known CE-modifying variants in the gene *UNC13A* (Table S7); therefore, these variants were not recovered in SpliPath analyses. The challenge of predicting these variants likely relates to the fact that they induce *UNC13A* CE retention only if TDP-43 is dysfunctional. Conversely, it is possible that the observed *CEP290* CE enhancing variant induces more constitutive “TDP-43-independent” CE retention that makes it easier for models to predict. Therefore, improvements in underlying AI prediction models will be required to comprehensively catalog all crsQTLs for TDP-43 CEs in ALS. However, our results nonetheless provide a proof of principle that SpliPath can be used to discover crsQTLs in patient WGS that map to splice junctions from artificial laboratory models.

## Discussion

Studies of simple Mendelian disorders can leverage familial segregation patterns to discover causal splice-altering mutations, while studies of polygenic traits can leverage bulk RNA-seq reference panels and GWASs to discover disease-associated sQTLs.^13^^,^^23^ However, it is more challenging to identify disease associations mediated by splice-altering variants that are both rare and not associated with Mendelian disease inheritance. In this study, we developed SpliPath crsQTL analyses as a new approach to address this challenge. We also compared the value of our approach with respect to a simpler SpliceAI-informed BT strategy. Our core hypothesis was that SpliPath would exhibit superior performance for genes where only a specific subset of splice-altering genetic variants was observed in the population are pathogenic. Conversely, we expected that SpliceAI-informed BTs would perform better in scenarios where most observed splice-altering variants are pathogenic. A key step in our framework was the use of sequence-to-function AI models, including SpliceAI and Pangolin, to link rare variants from a genetic dataset with a filtered set of reference splice junctions for crsQTL clustering. This hypothesis and approach were substantiated within our test case analyses of ALS datasets. We expect that our methods, results, and conclusions will be readily generalizable to any trait with a contribution from rare genetic variation and where disease-relevant transcriptomics datasets are available to be leveraged for the assembly of crsQTLs. Examples within the field neurology, including the multi-omics data repository from Accelerating Medicines Partnership in Alzheimer's disease and in Parkinson's disease (AMP-AD^24^ and AMP-PD^25^).

Numerous studies have already explored using paired transcriptomics data to validate splice-altering variant predictions in Mendelian traits^13^^,^^23^; however, here we demonstrate use of SpliPath to cluster variants based on splice junction matches within either paired or unpaired RNA-seq datasets. The capacity to leverage unpaired datasets is particularly relevant for disorders such as ALS, where disease-relevant tissues are inaccessible in living patients. This challenge makes it difficult to scale the generation of paired datasets to very large cohorts. With SpliPath, it is instead possible to use a moderately sized reference dataset, such as the NYGC cohort, to annotate crsQTLs in notably larger WGS-only datasets. We also highlight that our SpliPath approach provides an interesting opportunity to directly annotate large WGS datasets using RNA-seq of artificial laboratory models as the reference datasets. This breaks with the traditional convention in GWAS sQTL studies that RNA-seq of human cohorts is the required reference for human genetics analyses. In this study, the laboratory model we focused on was TDP-43 depletion in induced pluripotent stem cell-derived neurons. We selected this system because it recapitulates the most significant known neuropathological process in ALS while avoiding challenges in sensitivity, technical noise, and biological noise that occur with patient tissue.^21^^,^^22^ Furthermore, the prior discovery of *UNC13A* CE modifier variants raised the question of whether additional genetic variants also regulate toxic CE retention events in patients. We provided a proof of principle for this strategy with the discovery and validation of a crsQTL for a *CEP290 CE* retention event that is also induced by TDP-43 dysfunction. Elucidating the biological relevance of the crsQTL identified in this study will require further study and future research; however, our approach for ALS again provides a clear analytical template that could readily be reused for other disorders.

An incidental but noteworthy observation in our study was the added gains for extending SpliceAI predictions beyond the 50 bp windows considered during the generation of widely used “precomputed scores.” Specifically, we observed that extending the SpliceAI search window from 50 to 500 bp allowed for the discovery of a first-of-its-kind ALS intronic mutation hotspot. This occurred at a predicted branchpoint for the *KIF5A* gene and was validated using a minigene assay. As *KIF5A* is already being explored as a target for splice-altering antisense oligonucleotide therapies,^26^ the discovery of such variants may soon have important clinical relevance. In this case, extending SpliceAI prediction windows for targeted analyses of known disease genes is relatively trivial, but recomputing SpliceAI scores at the genome-wide scale for large cohorts involves substantial computational overhead. In cases where this computational overhead is prohibitive, SpliPath can provide one mechanism to prioritize a genomic search where disease-relevant crsQTL are most likely to be discovered.

Finally, we highlight that our functional clustering strategy can be extended beyond splicing to other molecular phenotypes that influence gene expression and regulation. For instance, in collapsed promoter QTL analysis, promoter activity data from analyzing disease-relevant cap-analysis gene expression sequencing (CAGE-seq) dataset or assay for transposase accessible chromatin (ATAC-seq) dataset could be used as reference datasets to group variants with predicted impact on promoter function, with models like Sei^27^ providing the predictions. Similarly, in collapsed polyadenylation QTL analysis, polyadenylation site usage from analyzing disease-relevant long-read isoform sequencing (Iso-seq) datasets could inform the grouping of variants affecting transcript stability and processing. Here, models like APARENT^28^ could predict variant effects on polyadenylation efficiency. By applying this strategy across multiple molecular processes, we can create a broader framework for clustering impactful rare variants, further enhancing the discovery of disease-relevant regulatory elements.

### Limitations of the study

Despite the demonstrated value of SpliPath as a complement to traditional BT analyses, users should be aware of several important limitations. First, SpliPath is less powerful than SpliceAI-informed BTs in scenarios where the ratio of pathogenic to benign splice-altering variants is high. We demonstrated this with *NEK1* in ALS, but this scenario is expected to be common in diseases where loss-of-function toxicity is the primary driver of risk. In such cases, SpliceAI-informed BTs are well suited to detect disease-associated genes. However, SpliceAI-informed BTs struggle in the opposite scenario, where pathogenic splice-altering variants are diluted by non-pathogenic ones, precisely where SpliPath excels. Given these strengths, we recommend using SpliPath as complement to BTs rather than a replacement.

The second limitation of SpliPath is its dependence on the completeness and resolution of reference transcriptomics datasets used to build crsQTLs. Comprehensive sampling of all rare splice events requires larger sample sizes than needed for comprehensive sampling of common splice junctions. This is important, as there is a higher risk for missing relevant associations in studies where reference cohorts are too small. To illustrate this, we modeled the probability of detecting at least one sample expressing a splice junction across varying rarity and cohort size (Figure S7). These analyses highlight that, while our study successfully recovered an ALS splice defect, which is expected to be present in approximately 0.08% of patients (9 distinct variants with a cumulative patient carrier frequency of 0.08% across 12,905 patients), current sample sizes within the NYGC reference cohort suggest that more events in this and rarer frequency bins likely remain to be discovered by future studies that extend our work to larger patient cohorts. For studies with limited reference datasets, it can therefore again be desirable to perform SpliPath analyses alongside traditional burden analyses to leverage the complementary strengths of both approaches. Similarly, although bulk RNA-seq reference datasets have been very effective for a wide range of sQTL analyses, single-cell sequencing can provide greater power to detect additional cell-type-specific events.^29^ Widely used platforms like 10× prioritize 3′ end sequencing, making them unsuitable for comprehensive splice junction analysis, but multiple full-length single-cell RNA sequencing (scRNA-seq) technologies are available.^30^^,^^31^ As with common variant sQTL studies, we therefore envisage that scRNA-seq will provide greater power for future crsQTL analyses.

Finally, it should be noted that the performance of SpliPath is tightly coupled to the performance of underlying sequence-to-function AI models. Our work primarily focuses on SpliceAI, as this is the most widely used tool for splice prediction analyses. However, during review, it was highlighted that our ALS use case might be better served by models that leverage brain-specific training datasets. We therefore also updated SpliPath to be compatible with Pangolin (Figure S6) and included both Pangolin and SpliceTransformer in comparison benchmark analyses of the *KIF5A* gene. For splice events that cannot be reliably modeled using these tools, including intronic ALS-associated SNPs in *UNC13A*, SpliPath will be unable to build crsQTLs. We will therefore continue to update SpliPath so that the tool remains readily compatible with the more advanced splice prediction tools in the future.

## Resource availability

### Lead contact

Requests for further information and resources should be directed to the lead contact, Kevin Kenna (k.p.kenna@umcutrecht.nl).

### Materials availability

This study did not generate new unique reagents.

### Data and code availability

• This paper analyzed existing, publicly available RNA-seq data from the New York Genome Center ALS consortium (GEO: GSE137810). WGS data from Project MinE are available upon request (https://projectmine.com/).
• All original code has been deposited at https://github.com/KennaLab/SpliPath and is publicly available. An archival version is listed in the key resources table.
• Any additional information required to reanalyze the data reported in this paper is available from the lead contact upon request.

## Consortia

The members of the Project MinE ALS Sequencing Consortium are Philip van Damme, Philippe Corcia, Philippe Couratier, Patrick Vourc’h, Orla Hardiman, Russell L. McLaughin, Marc Gotkine, Vivian Drory, Nicola Ticozzi, Vincenzo Silani, Jan H. Veldink, Leonard H. van den Berg, Mamede de Carvalho, Marta Gromicho, Jesus S. Mora Pardina, Monica Povedano, Peter M Andersen, Markus Weber, Nazli A. Başak, Ammar Al-Chalabi, Christopher E. Shaw, Pamela J. Shaw, Karen E. Morrison, John E. Landers, Jonathan D. Glass, and Clifton L. Dalgard.

The members of the NYGC ALS Consortium are Hemali Phatnani, Justin Kwan, Dhruv Sareen, James R. Broach, Zachary Simmons, Ximena Arcila-Londono, Edward B. Lee, Vivianna M. Deerlin, Neil A. Shneider, Ernest Fraenkel, Lyle W. Ostrow, Frank Baas, Noah Zaitlen, James D. Berry, Andrea Malaspina, Pietro Fratta, Gregory A. Cox, Leslie M. Thompson, Steve Finkbeiner, Efthimios Dardiotis, Timothy M. Miller, Siddharthan Chandran, Suvankar Pal, Eran Hornstein, Daniel J. MacGowan, Terry Heiman-Patterson, Molly G. Hammell, Nikolaos. A. Patsopoulos, Oleg Butovsky, Joshua Dubnau, Avindra Nath, Robert Bowser, Matthew Harms, Eleonora Aronica, Mary Poss, Jennifer Phillips-Cremins, John Crary, Nazem Atassi, Dale J. Lange, Darius J. Adams, Leonidas Stefanis, Marc Gotkine, Robert H. Baloh, Suma Babu, Towfique Raj, Sabrina Paganoni, Ophir Shalem, Colin Smith, Bin Zhang, Thomas Blanchard, Brent Harris, Iris Broce, Vivian Drory, John Ravits, Corey McMillan, Vilas Menon, Lani Wu, Steven Altschuler, Yossef Lerner, Rita Sattler, Kendall Van Keuren-Jensen, Orit Rozenblatt-Rosen, Kerstin Lindblad-Toh, Katharine Nicholson, Peter Gregersen, Jeong-Ho Lee, Matt Brauer, Shameek Biswas, Kimberly A Wilson, Sulev Koks, Stephen Muljo, Bryan J. Traynor, Robert Moccia, Seng Cheng, Andrew Deubler, Giovanni Coppola, Mickey Atwal, Michael Cantor, William Salerno, Eli Stahl, Matt Anderson, David Frendewey, Daphne Koller, and Mary Rozenman.

## Acknowledgments

We are grateful to the patients with ALS and their families for allowing donation of tissue for research. K.K. is supported by grants from the 10.13039/501100003246Dutch Research Council (ZonMW-VIDI 91719350) and the 10.13039/501100014076ALS Foundation Netherlands. K.K., E.A., and J.H.V. are supported by ALS Stichting (grant ALS Tissue Bank – NL). All NYGC ALS Consortium activities are supported by the 10.13039/100000971ALS Association (ALSA; 19-SI-459) and the 10.13039/100013352Tow Foundation. This project has received funding from the 10.13039/501100000781European Research Council under the European Union’s Horizon 2020 Research and Innovation Programme (grant agreement no. 772376 – EScORIAL).

## Author contributions

Conceptualization, Y.W. and K.K.; methodology, Y.W. and K.K.; investigation, Y.W.; minigene reporter assay, C.v.D. and I.T.; writing – original draft, Y.W. and C.v.D.; writing – review & editing, Y.W., J.H.V., and K.K.; funding acquisition, K.K.; resources, P.H., B.K., M.K., E.A., R.J.P., L.H.v.d.B., J.C.-K., and J.H.V., Project MinE ALS Sequencing Consortium, and NYGC ALS Consortium; supervision, J.H.V. and K.K.

## Declaration of interests

J.H.V. reports sponsored research agreements with Biogen, Eli Lilly, and Astra Zeneca. R.J.P. reports sponsored research agreements with Amylyx. L.H.v.d.B. has served on advisory boards for Biogen, Amylyx, Ferrer, Corcept, QurAlis, Cytokinetics, Argenx, VectorY, Verge, and Nura Bio.

## STAR★Methods

### Key resources table

**Table:** 

REAGENT or RESOURCE	SOURCE	IDENTIFIER
**Chemicals, peptides, and recombinant proteins**		

DMEM/F-12, GlutaMAX™	Thermo Fisher Scientific	Ca. No. 31331028
10% fetal bovine serum	Tico Europe	Ca. No. FBSEU500
0.5% Penicillin-Streptomycin	Thermo Fisher Scientific	Ca. No. 15140122
Gibson Assembly kit	New England BioLabs	Ca. No. E5510S
Site directed mutagenesis kit	New England BioLabs	Ca. No. E0554S
Lipofectamine Transfection Reagent	ThermoFisher	Ca. No. L3000001
DAPI	N/A	N/A

**Deposited data**		

SpliPath	This paper	https://github.com/KennaLab/SpliPath, https://doi.org/10.5281/zenodo.16871553

**Experimental models: Cell lines**		

SH-SY5Y cells	N/A	N/A

**Oligonucleotides**		

Primers for minigene reporter assay, see Table S2	IDT	N/A

**Recombinant DNA**		

Plasmids pGEMT-PT2A-GFP-Tdtomato-iRFP670	Liu et al.^32^	RRID:Addgene_111812
Plasmids EF1alpha-mScarlet	This paper	N/A

**Software and algorithms**		

STAR (v2.7.3a)	Dobin et al.^33^	https://github.com/alexdobin/STAR
RegTools (v0.5.2)	Cotto et al.^34^	https://github.com/griffithlab/regtools
SpliPath	This paper	https://github.com/KennaLab/SpliPath, https://doi.org/10.5281/zenodo.16871553
LeafCutterMD	Li et al.,^35^ Jenkinson et al.^14^	https://davidaknowles.github.io/leafcutter/
Snaptron	Wilks et al.^36^	https://snaptron.cs.jhu.edu/
SpliceAI (v1.3)	Jaganathan et al.^10^	https://github.com/Illumina/SpliceAI
Pangolin (v1.0.1)	Zeng et al.^11^	https://github.com/tkzeng/Pangolin
SpliceTransformer (v1.0.0)	You et al.^17^	https://github.com/ShenLab-Genomics/SpliceTransformer
CrossMap	Zhao et al.^37^	https://github.com/liguowang/CrossMap
RVAT	Hop et al.^38^	https://github.com/KennaLab/rvat
R poolr	Cinar et al.^39^	https://cran.r-project.org/web/packages/poolr/index.html
FlowJo	BD Bioscience	https://www.flowjo.com/

**Other**		

Ensembl gene annotation release 98	https://www.ensembl.org/	Homo_sapiens.GRCh38.98.gtf
ENCODE blacklist	Amemiya et al.^40^	https://github.com/Boyle-Lab/Blacklist
gnomAD (v3.1.2)	Chen et al.^41^	https://gnomad.broadinstitute.org/

### Experimental model and study participant details

#### SH-SY5Y cells culture

SH-SY5Y cells were cultured in a 24 well plate at a density of 100,000 cells and 1 mL of medium (DMEM with GlutaMAX, 10% fetal bovine serum and 0.5% Penicillin-Streptomycin).

### Methods details

#### RNAseq and DNAseq datasets

This study used WGS from 2 ALS cohorts (Project MinE, NYGC ALS sequencing consortium) and RNAseq from 2 ALS relevant datasets (i^3^Neuron models of ALS related TDP-43 dysfunction^21^ and multi-tissue RNAseq of ALS patients by the NYGC ALS sequencing consortium). The Project MinE data includes WGS for 6,652 ALS patients and 2,472 controls. Full details of this cohort have been reported in van Rheenen al.^4^^,^^5^ Data from the NYGC ALS sequencing consortium includes full WGS for 294 ALS/motor neuron disease (MND) spectrum patients and 76 neurotypical controls, as well as paired RNAseq of up to 4 brain and spinal cord regions sampled from the same matched donor cohort (motor cortex, frontal cortex, cervical spinal cord, lumbar spinal cord, GEO:GSE137810). RNAseq data for TDP-43 knockdown/control i^3^Neurons were retrieved from the European Nucleotide Archive (ENA: PRJEB42763).^21^ For a secondary check for ALS related cryptic exons (Figure S5), we used RNAseq data for TDP-43 negative/positive neuronal nuclei from postmortem patient tissue, retrieved from the Gene Expression Omnibus (GEO: GSE126543).^42^ For validating the association between crsQTL and ALS, we used an ALS WXS cohort (Project MinE, manuscript in preparation) including 12,905 ALS patients and 69,718 controls.

#### Alignment and pre-processing of RNAseq and DNAseq datasets

All RNA-seq data was aligned to the GRCh38 reference genome using STAR v2.7.3a.^33^ transcript annotations were based on Ensembl version 98. To enhance capture of unannotated junctions in downstream analyses, STAR was used in two-pass mode (whereby unannotated junctions identified during first pass mapping are used to generate a refined splice junction annotation file for second pass mapping). All WGS data from the NYGC was aligned to the hg38 reference genome using BWA.^43^ Variant calling and individual genotyping were performed in accordance with best practices outlined for the Genome Analysis Toolkit (GATK v4.1.0).^44^ Processing of the WGS dataset from Project MinE is described by van Rheenen et al.^4^ and it is aligned to build GRCh37. 27 sample duplication events between Project MinE and the NYGC dataset were identified by using KING to analyze genetic relatedness.^45^ These relatedness analyses were conducted using a quality controlled set of common variant markers that were extracted from VCF files using plink v1.9.^46^ Quality control filters for the common variant markers included filtering for a set of biallelic LD pruned single nucleotide variants that exhibited a MAF>0.01, genotype call rate>0.9 and no evidence for deviation from Hardy Weinberg equilibrium (*p* < 0.001).

#### Outlier splice junction identification

RegTools v0.5.2^34^ was used to define candidate splice junctions based on the detection of split reads within the aligned RNAseq bam files. Split reads were only considered in the event that they mapped to the reference genome with a minimum overlap of 6bp at both ends. LeafCutterMD^14^^,^^35^ analysis was used to detect outlier splicing junctions of each tissue. This involves two steps: 1) LeafCutter intron clustering method was performed on the splicing junction files, with parameters: minimum 10 reads in a cluster, minimum 0.0001% of reads in a cluster that support a junction, and maximum intron length of 500kbp; 2) LeafCutterMD analysis on the clustered introns with parameters: maximum 500 introns in a cluster and minimum 10 reads mapped to a cluster. The junctions with an outlier *p* value <0.05 were considered as outlier junctions. The outlier splicing junctions were classified as being “annotated” or “unannotated” by comparison to reference splice junction coordinates retrieved from Ensembl 98 and the Snaptron database.^36^ Unannotated splice junctions were classified as being either unannotated exon skipping junction, unannotated donor junction, or unannotated acceptor junction. For designation as an unannotated exon skipping, we required that the data supported skipping of annotated exons without creating new unannotated donor/acceptor sites. For designation as an unannotated donor/acceptor junction, we required that an observed unannotated donor/acceptor site was paired with an annotated splice site. More complex splice aberrations involving multiple genes or multiple unannotated splice sites were regarded as low confidence events and excluded from our analyses in this study. Candidate junctions occurring within ENCODE blacklist regions, genes with median expression TPM <2 or sex chromosomes were also excluded.^40^ To obtain disease-relevant splice junctions of higher confidence, we required that the outlier junctions from analysis of NYGC RNAseq dataset were reproducible across RNAseq profiles of at least 2 tissue types from the same donor. For each outlier junction, Fisher’s method in *poolr* package^39^ was used to combine the tests of multiple tissues.

#### Obtaining reference junctions from TDP43-dependent cryptic exons

The differential splicing analysis results for TDP43-KD i^3^Neurons and controls were retrieved from the supplemental material of Brown et al.^21^ The corresponding splicing junctions of the cryptic exons were those labeled as cryptic with FDR <0.05. For these junctions, we examined their expression in RNAseq profiles of postmortem brain and spinal cord in NYGC cohort. The reference junctions for crsQTL analysis were those that were detectable (read count ≥ 1) in any patient’s tissue but not detectable in tissue of more than 10% of controls.

#### Prediction of splice-altering consequences for DNA variants

We annotated DNA variants with predictions scores from SpliceAI v1.3.^10^ We scored the predicted effect of every observed minor allele on potential splice sites located within 500bp of the variant position. For the two CE modifier variants in *UNC13A*, the effects were scored on potential splice sites located within 4999bp of the variants. The final SpliceAI score was taken as the maximum delta score predicted for any given donor gain, donor loss, acceptor gain, and acceptor loss event. DNA variants were only considered as potentially splice-altering in the event of a SpliceAI score ≥0.2. To compare the efficacy of crsQTL-informed disease association testing with gene burden testing, DNA variants were also annotated using SpliceAI -D4999 (which predicts potential splice sites within 4,999 bp of the variant position), Pangolin v1.0.1^11^ with the setting –distance = 5000 and SpliceTransformer v1.0.0.^17^ DNA variants were considered as splice-altering when the absolute Pangolin score ≥0.2, or when SpliceTransformer score >0.27 and the effect was not specific to non-neuronal tissues. The SpliceAI input genome annotation file was derived from Ensembl version 98, including only transcripts labeled as ‘basic’. The input genome annotations files for Pangolin and SpliceTransformer was GENCODE version 32, which corresponds to Ensembl version 98. For DNA variants in Project MinE, the variant coordinates were first lifted from GRCh37 to GRCh38 using CrossMap^37^ and then annotated using the prediction tools.

#### Matching splice-altering variants with reference junctions

For every splice junction, we restricted our DNA variant search space to the full length of the exon at the nearest annotated 5′ donor site, the full length of the exon at the nearest annotated 3′ accepter site, and the entirety of the intervening sequence. SpliceAI generates four predictions for the effects of a genetic variant: donor site gain, donor site loss, acceptor site gain, and acceptor site loss. The criteria for 'matching' a SpliceAI prediction with a reference junction are as follows: 1) If SpliceAI predicts the gain of an unannotated donor/acceptor site, a match is considered when a junction uses this unannotated site; 2) If SpliceAI predicts the loss of an annotated donor/acceptor site, a match is considered when a junction omits this annotated site. Predictions that exceed a delta score threshold of 0.2 were used for matching with reference splicing junctions. In most instances, multiple SpliceAI predictions can pass the threshold for a single genetic variant. Since SpliceAI is able to predict the probabilities at up to four donor and acceptor sites, it is possible that a splice junction does not fully align with all predictions. We therefore classify a splice junction as a “partial match” if it aligns with some of a variant’s predictions, and as a “full match” if it aligns with all predictions (detailed Figure S1). Pangolin generates two predictions: increased splice site usage and decreased splice site usage. Similarly, the matching criteria is 1) If Pangolin predicts the increased usage of an unannotated donor/acceptor site, a match is considered when a junction uses this unannotated site; 2) If Pangolin predicts the decreased usage of an annotated donor/acceptor site, a match is considered when a junction omits this annotated site. Splice-altering variants that are full or partial matches to the same reference junction are grouped together during crsQTL analyses. To match the splice-altering variants with the outlier splicing junctions in the paired NYGC WGS and RNAseq datasets, we restricted the analysis to genetic variants that exhibited a miner allele/homozygous carrier frequency below 0.1% among gnomAD (v3.1.2)^41^ non-neurological disease controls.

#### Case-control association testing

Case-control association tests for genes (in BT analyses) and crsQTL were performed using the Rare Variant Analysis Toolkit (RVAT)^38^ (https://github.com/KennaLab/rvat). Firth penalized logistic regression was used to model case-control status with respect to rare variant burden. All association analyses of the Project MinE WGS cohort included the following covariates; sex, sequencing platform and the first 4 principal components derived from PCA of common variant profiles. Association analyses of the Project MinE WXS cohort included sex, 10 principal components and the burden of synonymous variants under 0.1% allele frequency. Rare variants were only included for association testing if carrier frequency within the cohort was below 0.1%,^6^^,^^7^ under either an allelic or recessive genetic model. Genomewide rare variant association screens were performed under both allelic and recessive model.

#### *EPG5* splice junction usage and variant dosage association testing

The association between the *EPG5* splice junction usage (quantified as the -log_10_ of LeafCutterMD outlier *p* value) and the dosage of variant NC_000018.10:g.45903935T>TTCAC was tested using linear regression. Sex, RIN, RNAseq platform, and proportions of ancestry components were included as covariates.

#### SpliPath package

The SpliPath R package was developed to support reuse of our workflow for any desired combination of input WGS and RNAseq datasets. The tool includes functions to use output predictions from either SpliceAI or Pangolin during crsQTL discovery. The SpliPath package also includes shiny browsers to visualize and manually inspect candidate crsQTL in detail. We have developed one browser tailored to paired DNA-RNAseq datasets, where RNAseq and WGS data are both derived from the same donor (example browser: https://yanwang271.shinyapps.io/splipath_browser/). We have also developed a more lightweight browser for unpaired DNA-RNAseq datasets, where input WGS and RNAseq datasets are not derived from the same donor subjects (example browser: https://yanwang271.shinyapps.io/splipath_browser_unpaired_analysis/). The SpliPath R package can be installed from https://github.com/KennaLab/SpliPath. The make_sashimi_like_plot and make_gene_wise_plot function in SpliPath were adapted from make_differential_splicing_plot function in LeafCutter.^35^ The analyses in this study were performed using R 3.6.

#### *KIF5A* minigene reporter assay

The *KIF5A* minigene reporter construct (Figure S4) was based on the design described by Cheung et al.^16^ Three branchpoint mutations (NM_004984.4:c.2993-58A>C, c.2993-58A>G, c.2993-58A>T) and two control mutations were tested for their exon-skipping effects. The negative control variant (c.2993-12T>C) was selected from the gnomAD (v3.1.2)^41^ database where it exhibited an allele count = 5 in the non-neurological disease group. The positive control variant (c.3020 + 3A>G) was selected as a splice consensus region variant that we reported previously^8^ (Table S2). Vector assembly was performed using the Gibson Assembly kit from NEB (Ca. No. E5510S, New England BioLabs)^47^ and primers 1–12 (Table S2). Sequences were obtained from the plasmids pGEMT-PT2A-GFP-Tdtomato-iRFP670 for the bacteria backbone^32^ and an in-house plasmid containing the EF1alpha-mScarlet and polyA tail sequences. The *KIF5A* minigene sequence was extracted from genomic DNA derived from SH-SY5Y cells. The branchpoint mutations and control mutations were introduced using the site directed mutagenesis kit from NEB and primers 13–21 (Table S2) (Ca. No. E0554S, New England BioLabs).^48^ Construct sequences were validated using Sanger sequencing. SH-SY5Y cells were cultured in a 24 well plate at a density of 100,000 cells and 1 mL of medium (DMEM with GlutaMAX, 10% fetal bovine serum and 0.5% Penicillin-Streptomycin). Cells were transfected with 100 ng of plasmid using the Lipofectamine Transfection Reagent (Ca. No. L3000001, ThermoFisher) as per manufacturer instructions. After 48 h of incubation, cells were harvested and stained with DAPI to select live cells. Fluorescence was measured on a BD FACSCanto II Flow Cytometer. Gates were set to select living and single cells using untransfected cells. A compensation protocol was set up using cells transfected with in house plasmids containing eGFP or mScarlet only. The data was analyzed using the FlowJo software. mScarlet signals were obtained from eGFP positive cells to ensure transfection. The exon skipping signal was defined as the median mScarlet signal averaged over 3 technical replicates divided by the median mScarlet signal averaged over 3 technical replicates of the wildtype plasmid. The experiments were repeated 4 times. One-way ANOVA followed by T tests (adjusted for multiple testing using *Benjamini–Hochberg* false discovery rate method) were performed to compare the exon skipping signals of wildtype insertions to that of negative control, positive control, and target variants insertions.

#### Power analysis

Power analyses were performed to estimate the probability that a reference RNAseq cohort includes at least one donor that expresses a specific disease relevant splice junction. Assuming a Poisson distribution, the probabilities were calculated using the *ppois* function in R stats package v4.4. Probabilities were estimated for expected proportion of patients expressing a given splice junction of 0.0008, 0.001, 0.005, 0.01, 0.05, and 0.1 and sample sizes of 300, 500, 1000, 5000, and 10000 donors.

### Quantification and statistical analysis

Detailed procedures are described in the sections above. No additional statistical tests were performed.

## Supplemental information

Document S1. Figures S1–S7

Table S1. crsQTL identified from paired NYGC WGS and RNA-seq of patient tissues, related to Figure 2 Each row represents a variant mapped to an outlier splicing junction observed in an individual’s genome and tissues. Group: the disease classification of the individual. Coordinates_of_unannotated_junc: Genomic coordinates of the outlier splice junctions. Event: the splicing event annotation for the outlier splice junction. LeafCutterMD_Pval_X: the LeafCutter P values of the outlier splice junctions observed in different tissues. LeafCutterMD_Pval_meta: the meta P value combines the LeafCutterMD P values of multiple groups, calculated by Fisher’s method. DNA_variant: genomic location and allele information of the variants, formatted as “chromosome:position:reference allele:alternative allele”. Variant_dosage: the dosage of the variant of the individual. SpliceAI_score: the maximum SpliceAI delta score of the variant. SpliceAI_pred_match_junction: the type of ‘matching’ between a variant and a splice junction.

Table S2. The KIF5A exon 27-skipping variants tested in the minigene reporter assay and primers to assemble the vectors, related to Figure 2F

Table S3. crsQTL identified from Project MinE WGS dataset using NYGC RNAseq as reference, related to Figure 3B Each row represents a variant in Project MinE mapped to one of the 755 junctions involved in crsQTLs from NYGC dataset. Coordinates_of_unannotated_junc: Genomic coordinates of the splice junctions, formatted as “chromosome:start site:end site:strand”. DNA_variant_GRCh38: Genomic location and allele information of the variant in GRCh38, formatted as “chromosome:position:reference allele:alternative allele”. MinE_DNA_variant_GRCh37: Genomic location and allele information of the variant in the Project MinE dataset, aligned to GRCh37. SpliceAI_pred_match_junction: the type of ‘matching’ between a variant and a splice junction. Genetic_model: allelic or recessive model for counting the number of minor allele. case_minor_allele_count / ctrl_minor_allele_count: the number of minor alleles in the case or control group.

Table S4. crsQTL case and control association testing and conventional SpliceAI-informed gene burden testing in the Project MinE WGS cohort, related to Figure 3 and Table 2 ST4A: Each row represents a test for an identified crsQTL in Project MinE WGS cohort. Coordinates_of_unannotated_junc: genomic coordinates of the splice junctions, to which the crsQTL mapped. Analysis: allelic or recessive genetic model for association testing, and whether INDELs are included for testing. Nvar / Case_carriers / Ctrl_carriers: number of unique crsQTL variants found in number of case carriers or control carriers. P / Odds_ratio: P value and odds ratio of association testing using firth logistic regression. ST4B: Each row represents a burden test for SpliceAI-predicted splice-altering variants within a gene. Column definitions are identical to those in ST4A.

Table S5. KIF5A exon 27-skipping crsQTLs identified by SpliPath and splice-altering variants predicted by SpliceAI precomputed scores in Project MinE WGS and WXS cohort, related to Figure 3B and Table 1 ST5A: Each row is a crsQTL variant mapped to *KIF5A* exon 27 skipping, including SNVs and indel. DNA_variant_GRCh38: Genomic location and allele information of the variant in GRCh38, formatted as “chromosome:position:reference allele:alternative allele”. MinE_DNA_variant_GRCh37: Genomic location and allele information of the variant in the Project MinE WGS dataset, aligned to GRCh37. Case_carriers / Ctrl_carriers: number of carriers in case or control group. spliceaiXX: SpliceAI predictions scores for the variant (DS: delta score, DP: delta position, DG: donor gain, DL: donor loss, AG: acceptor gain, AL: acceptor loss). ST5B: Each row is a variant of SpliceAI precomputed scores ≥0.2. Column definitions are identical to those in ST6A.

Table S6. crsQTLs identified for TDP-43-associated CEs occurring in ALS cell models, related to Figure 5 ST6A: The crsQTL variants were identified in NYGC WGS dataset and Project MinE WGS dataset. Each row represents a variant mapped to an CE-relevant splicing junction. Coordinates_of_unannotated_junc: Genomic coordinates of the CE-relevant splice junctions, formatted as “chromosome:start site:end site:strand”. DNA_variant_GRCh38: Genomic location and allele information of the variant in GRCh38, formatted as “chromosome:position:reference allele:alternative allele”. MinE_DNA_variant_GRCh37: Genomic location and allele information of the variant in the Project MinE dataset, aligned to GRCh37. spliceaiXX: SpliceAI predictions scores for the variant (DS: delta score, DP: delta position, DG: donor gain, DL: donor loss, AG: acceptor gain, AL: acceptor loss). Mine_case_carrier / Mine_ctrl_carrier / NYGC_case_carrier / NYGC_ctrl_carrier: The number of variant carriers in case and control groups from the Project MinE and NYGC datasets, respectively. ST6B: Case-control association test of pooled TDP-43 CE crsQTL variants. Rare_variants_with_less_than_x_perct_carrier: the TDP-43 CE crsQTL variants with less than x% carrier were pooled for case and control association testing. Case / Ctrl: the total number of case or control samples in the NYGC and Project MinE WGS cohorts. Case_ carrier_frequency / Ctrl_carrier_frequency: the percentage of individuals carried any of the pooled variants in case or control groups. fisher_exact_P / fisher_exact_odds_ratio: the P value and odds ratio of fisher exact tests on minor allele frequency between two groups.

Table S7. Splice-altering predictions for two UNC13A CE modifier variants

Document S2. Article plus supplemental information
