# Supplementary material for: SpliPath enhances disease gene discovery in case-control analyses of rare splice-altering genetic variants
Source: Cell Rep Methods. 2025 Sep 17;5(10):101176. doi: 10.1016/j.crmeth.2025.101176 (PMC12570325; doi:10.1016/j.crmeth.2025.101176)
Supplement: Document S1. Figures S1–S7 [file mmc1.pdf]

Cell Reports Methods, Volume *5*

Supplemental information

SpliPath enhances disease gene discovery in case-control analyses of rare splice-altering genetic variants

Yan Wang, Charlotte van Dijk, Ilia Timpanaro, Paul Hop, Brendan Kenna, Maarten Kooyman, Eleonora Aronica, R. Jeroen Pasterkamp, Leonard H. van den Berg, Johnathan Cooper-Knock, Project MinE ALS Sequencing Consortium, NYGC ALS Consortium, Jan H. Veldink, and Kevin Kenna

Supplementary Figures

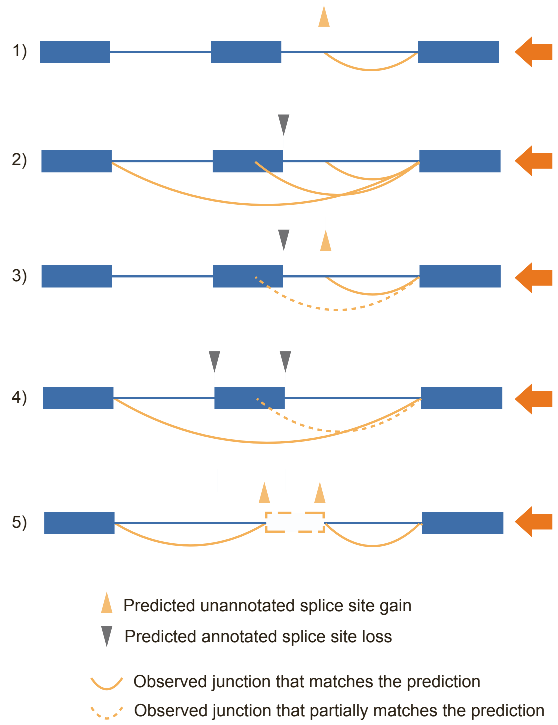

SpliceAI Prediction	Criteria for "Matched" Junction in RNAseq
Unannotated splice site gain	Unannotated junction that uses the predicted splice site.
Loss of annotated splice site	Unannotated junction that does use the predicted lost site*.
Loss of annotated splice site & gain of unannotated splce site	Unannotated junction that uses the predicted unannotated splice site, and unannotated junction that does not use the predicted lost site*.
Loss of two annotated splice sites	Unannotated junction that skips both of the predicted lost sites, and unannotated junction that skips one of the predicted lost site*.
Gain of two unannotated splice sites	Unannotated junction that uses the predicted unannotated splice site.

*The lost splice site can be replaced by an alternative annotated site or an unannotated site.

Figure S1. Criteria for matching SpliceAI variant effect predictions and observed splice junctions, related to Figure 1 and STAR Methods.
The orange arrowheads indicate the locations of predicted unannotated splice site gain events (delta scores ≥ 0.2) and the dark grey arrowheads indicate the position of predicted annotated splice sites loss events (delta scores ≥ 0.2). The solid orange arcs indicate RNAseq derived splice junctions that fully match a SpliceAI[1] prediction, and the dashed arcs indicate RNAseq derived splice junctions that partially match a SpliceAI prediction. The table specifies criteria for classifying the pairing of a SpliceAI prediction and RNAseq derived junction as "match". When SpliceAI predicts multiple splice sites gain/loss, the junctions match prediction of at least one but not all splice sites gain/loss are classified as "partial match".

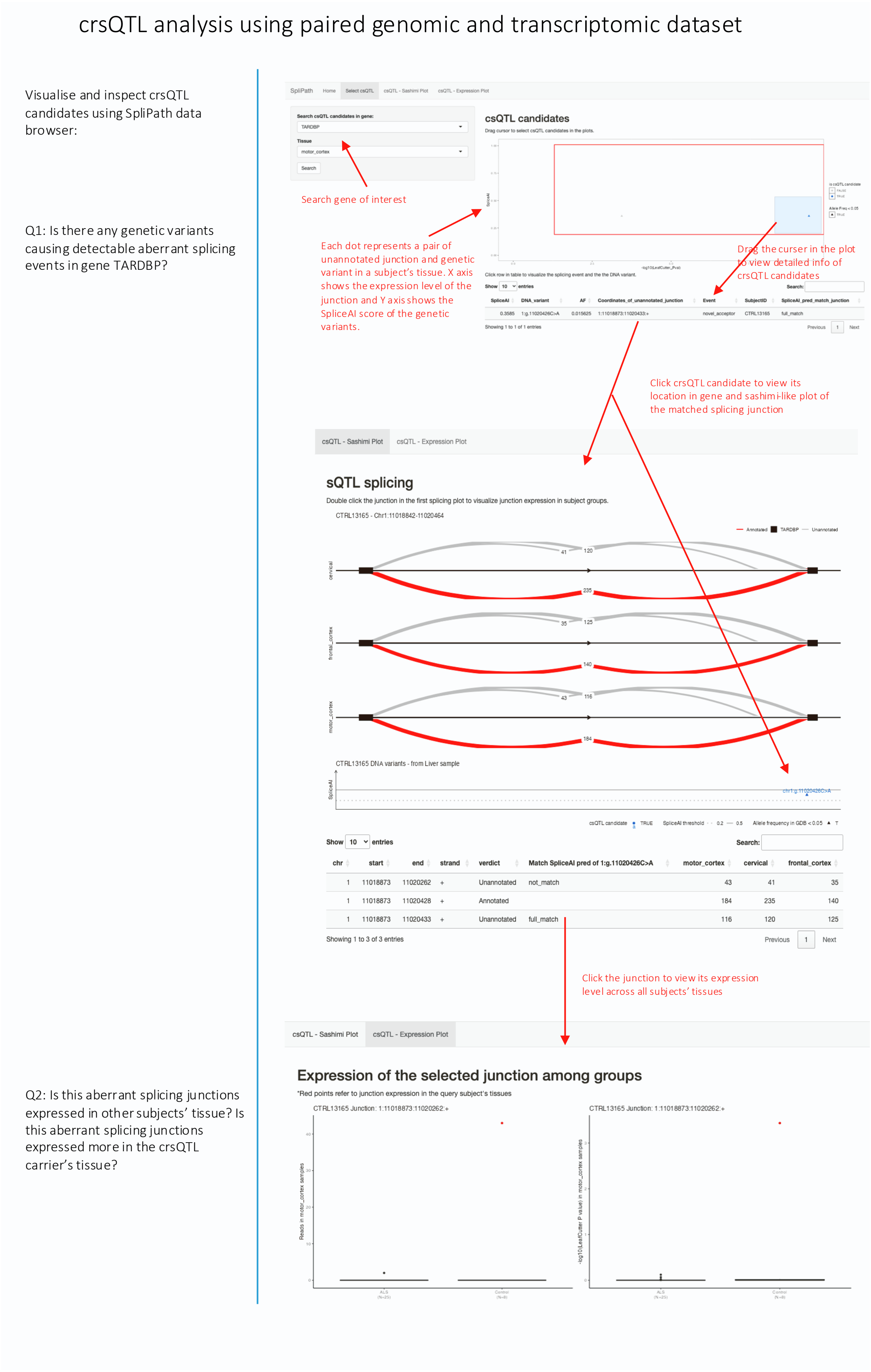

Figure S2: Overview of the built-in data browser within the SpliPath package, related to STAR Methods.

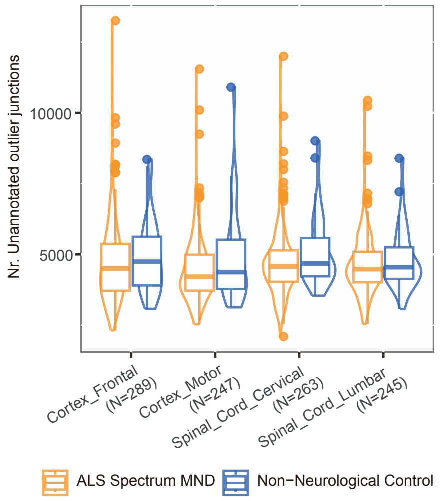

Figure S3. Summary of outlier splicing junction frequencies per individual in the paired RNAseq dataset from NYGC consortium, related to Figure 2.

Number of unannotated outlier splice junctions per individual identified by analyses of multi-tissue RNAseq profiles in the NYGC ALS cohort.

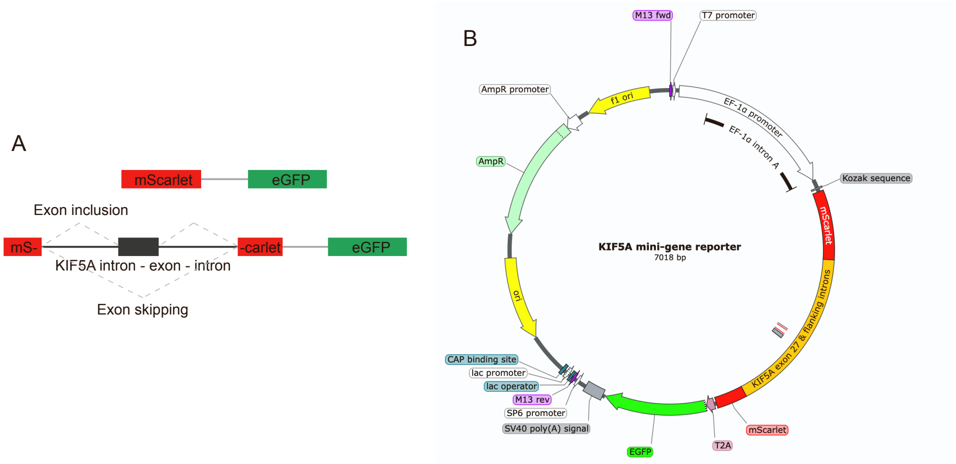

Figure S4. Design of minigene reporter assay, related to Figure 2F.

(A) Design of minigene reporter to validate the exon skipping effects of *KIF5A* SNVs at the predicted branchpoint. The plasmid contained an eGFP and a split mScarlet, into which the *KIF5A* intron 26 - exon 27 - intron 27 sequences were inserted.

(B) Design of the plasmid.

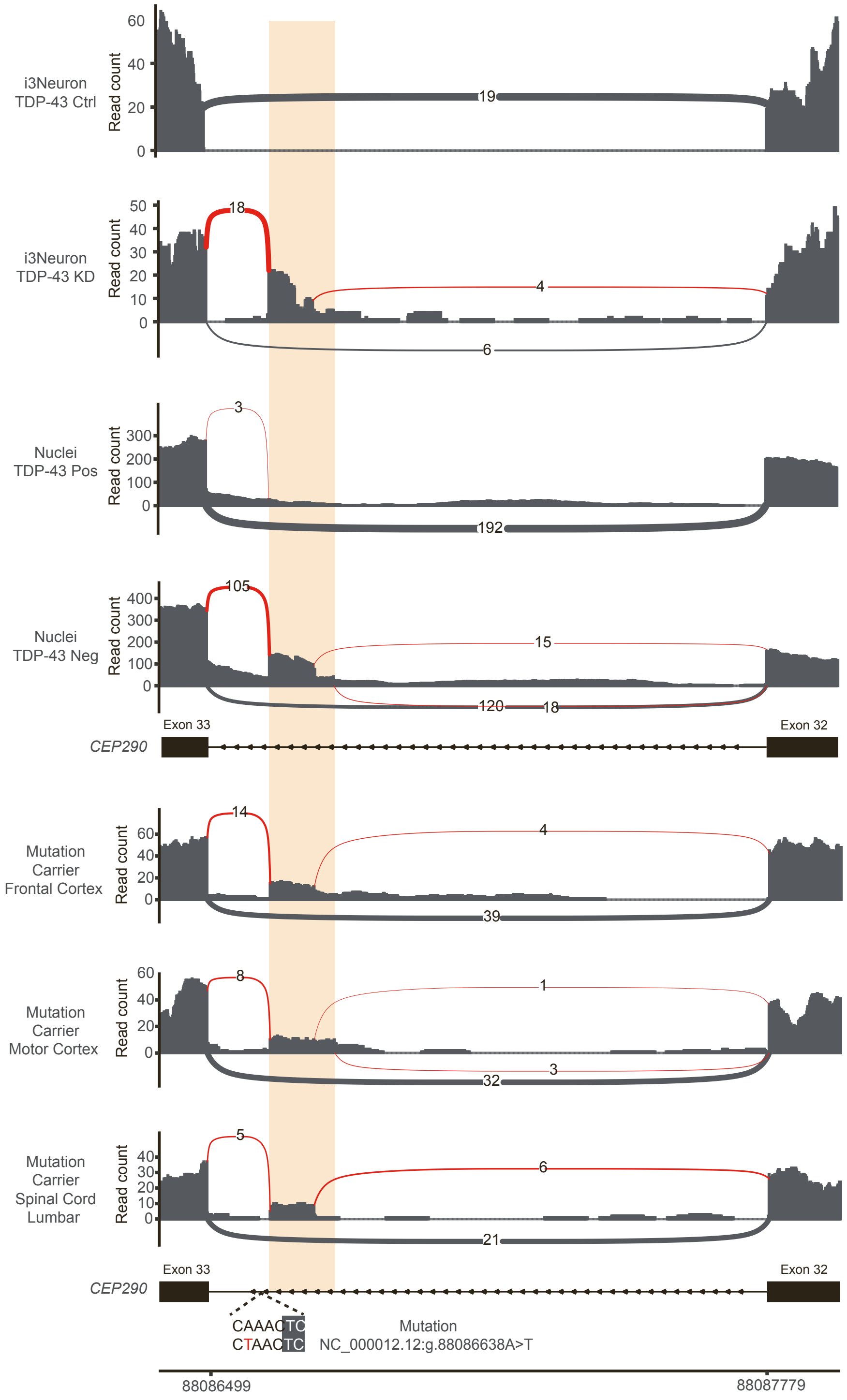

Figure S5. *CEP290* cryptic exon inclusion events were observed consistently across tissues within crsQTL variant carriers, related to Figure 5.

CEP290 cryptic exons were observed consistently across TDP43-KD i^{3}Neuron cells, sorted patient TDP43-negative nuclei (from GSE126543[2]), and brain and spinal cord samples of NC_000012.12:88086638A>T carrier.

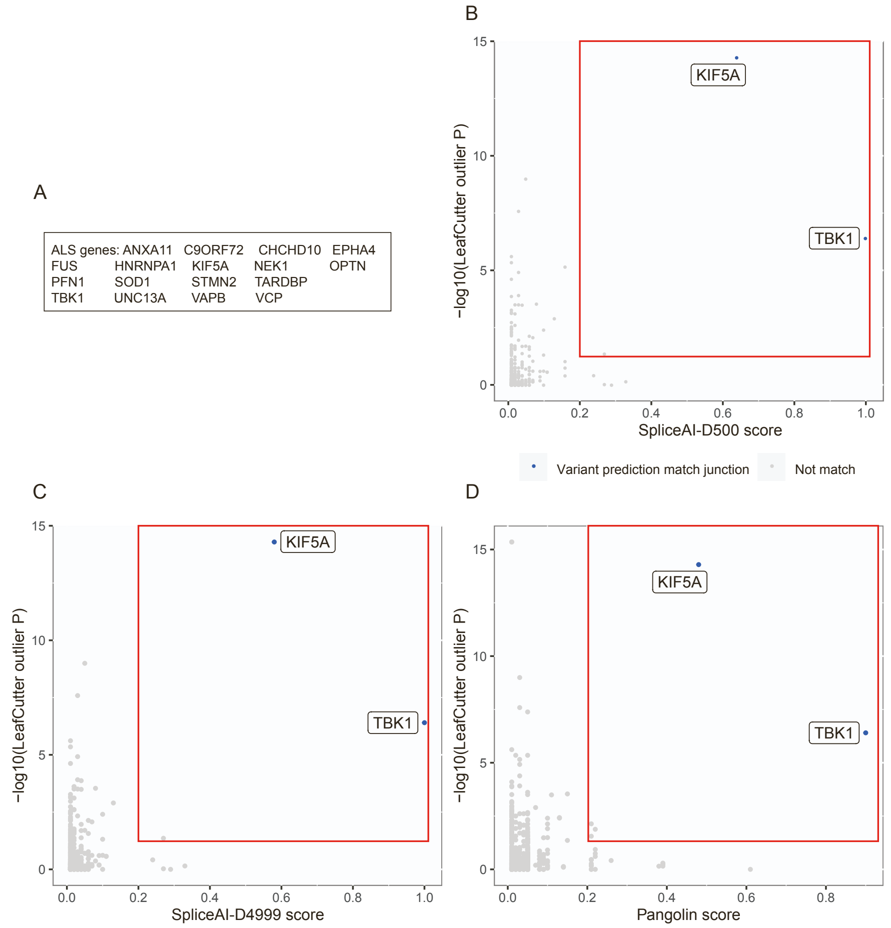

Figure S6. crsQTL retrieved for known ALS genes by applying SpliPath to the NYGC cohort with different variant prediction models, related to Figure 2 and STAR Methods.

(A) To compare the performance of nominating crsQTL with the application of SpliceAI-D500, SpliceAI-D4999 and Pangolin-D5000 models, we nominated crsQTL in the 17 ALS genes shown, using the paired NYGC WGS and RNAseq dataset.

(B, C and D) Scatter plot depicting tentative pairings of rare variants and splice junctions that occur within the same subgenic windows (Method) of known ALS genes in the same sample. The variant splice-altering scores from (B) SpliceAI-D500, (C) SpliceAI-D4999 and (D) Pangolin are shown in X axis and LeafCutter outlier P value of the splice junctions are shown in Y axis. Junction-variant pairings that fulfill SpliPath matching criteria are colored blue, whereas junction–variants pairings that do not fufill SpliPath matching criteria are colored grey. The red rectangles show the initial thresholds for matching: LeafCutter outlier P value < 0.05, SpliceAI ≥ 0.2, and Pangolin score ≥ 0.2. SpliPath identified the same crsQTL in *KIF5A* and *TBK1* by applying different prediction models. (B) is the same scatter plot with Figure 2C. It is shown here again to facilitate the comparison of results where different models were used.

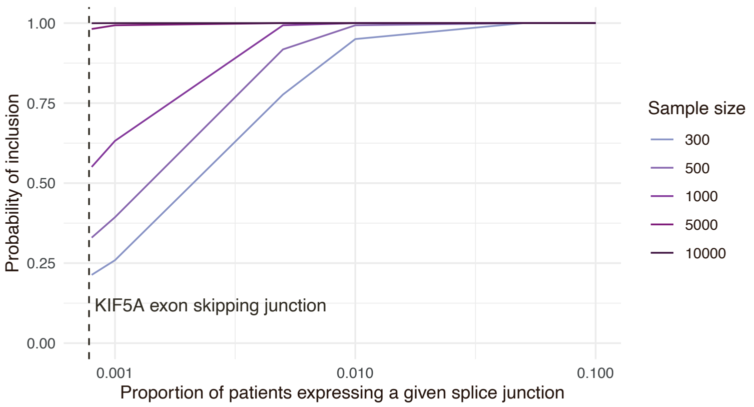

Figure S7. Probability of sampling a donor that expresses a given disease splice junction given varying expected proportion of patients expressing the junction and study sample sizes, related to STAR Methods.

The X axis is the expected proportion of patients expressing a given splice junction (Axis plotted on log10 scale). The Y axis is the probabilities of sampling at least one sample expressing that junction. The vertical dashed line shows the expected proportion of patients expressing the *KIF5A* exon skipping junction (0.0008). Each line represents and is colored by the sample size (See Methods).

Reference

1. Jaganathan, K., Kyriazopoulou Panagiotopoulou, S., McRae, J.F., Darbandi, S.F., Knowles, D., Li, Y.I., Kosmicki, J.A., Arbelaez, J., Cui, W., Schwartz, G.B., et al. (2019). Predicting Splicing from Primary Sequence with Deep Learning. Cell *176*, 535-548.e24. https://doi.org/10.1016/j.cell.2018.12.015.
2. Liu, E.Y., Russ, J., Cali, C.P., Phan, J.M., Amlie-Wolf, A., and Lee, E.B. (2019). Loss of Nuclear TDP-43 Is Associated with Decondensation of LINE Retrotransposons. Cell Rep *27*, 1409-1421.e6. https://doi.org/10.1016/j.celrep.2019.04.003.

Consortia

Project MinE ALS sequencing consortium

Philip van Damme[6,7], Philippe Corcia[8,9,10], Philippe Couratier[8,10], Patrick Vourc'h[9,11], Orla Hardiman[12,13], Russell L. McLaughin[14], Marc Gotkine[15], Vivian Drory[16], Nicola Ticozzi[17,18], Vincenzo Silani[17,18], Jan H. Veldink[2], Leonard H. van den Berg[2], Mamede de Carvalho[19,20], Marta Gromicho[19], Jesus S. Mora Pardina[21], Monica Povedano[22], Peter M Andersen[23], Markus Weber[24], Nazli A. Başak[25], Ammar Al-Chalabi[16,27], Christopher E. Shaw[26], Pamela J. Shaw[4], Karen E. Morrison[28], John E. Landers[29], Jonathan D. Glass[30,31], Clifton L. Dalgard[32]

6: KU Leuven - University of Leuven, Department of Neurosciences and Department of Neurology
7: VIB, Center for Brain & Disease Research, Leuven, Belgium.
8: Centre SLA, CHRU de Tours, Tours, France.
9: UMR 1253, iBrain, Université de Tours, Inserm, Tours, France.
10: Federation des Centres SLA Tours and Limoges, LITORALS, Tours, France.
11: Service de Biochimie et Biologie moléculaire, CHU de Tours, Tours, France
12: Academic Unit of Neurology, Trinity College Dublin, Trinity Biomedical Sciences Institute, Dublin, Republic of Ireland.
13: Department of Neurology, Beaumont Hospital, Dublin, Republic of Ireland.
14: Complex Trait Genomics Laboratory, Smurfit Institute of Genetics, Trinity College Dublin, Dublin, Republic of Ireland.
15: Neuromuscular Unit, Department of Neurology, Hadassah Medical Organization and Faculty of Medicine, Hebrew University of Jerusalem, Israel
16: Department of Neurology Tel-Aviv Sourasky Medical Centre , Israel.
17: Department of Neurology and Laboratory of Neuroscience, IRCCS Istituto Auxologico Italiano, Milano, Italy.

18: Department of Pathophysiology and Tranplantation, ‘Dino Ferrari’ Center, Università degli Studi di Milano, Milano, Italy.
19: Instituto de Fisiologia, Instituto de Medicina Molecular, Faculdade de Medicina, Universidade de Lisboa, Lisbon, Portugal
20: Department of Neurosciences, Hospital de Santa Maria-CHLN, Lisbon, Portugal.
21: ALS Unit, Hospital Universitario San Rafael, Madrid, Spain.
22: la Unitat Funcional de Motoneurona, Cap de Secció de Neurofisiologia, Servei de Neurologia, Hospital Universitario de Bellvitge-IDIBELL
23: Department of Clinical Science, Neurosciences, Umeå University, Sweden.
24: Neuromuscular Diseases Unit/ALS Clinic, Kantonsspital St. Gallen, 9007, St. Gallen, Switzerland.
25: Koç University, School of Medicine, KUTTAM-NDAL, Istanbul Turkey.
26: Maurice Wohl Clinical Neuroscience Institute, King's College London, Department of Basic and Clinical Neuroscience, London, UK.
27: Department of Neurology, King’s College Hospital, London SE5 9RS, UK
28: School of Medicine, Dentistry and Biomedical Sciences, Queen’s University Belfast, UK.
29: Department of Neurology, University of Massachusetts Chan Medical School, Worcester, MA, USA.
30: Department Neurology, Emory University School of Medicine, Atlanta, GA, USA.
31: Emory ALS Center, Emory University School of Medicine, Atlanta, GA, USA.
32: Department of Anatomy, Physiology & Genetics, The American Genome Center, Uniformed Services University of the Health Sciences, Bethesda, MD, USA

NYGC ALS Consortium

Hemali Phatnani[33], Justin Kwan[34,35], Dhruv Sareen[36], James R. Broach[37], Zachary Simmons[38], Ximena Arcila-Londono[39], Edward B. Lee[40], Vivianna M. Deerlin[40], Neil A. Shneider[41], Ernest Fraenkel[42], Lyle W. Ostrow[43], Frank Baas[44], Noah Zaitlen[45], James D. Berry[46], Andrea Malaspina[47], Pietro Fratta[48], Gregory A. Cox[49], Leslie M. Thompson[50], Steve Finkbeiner[51], Efthimios Dardiotis[52], Timothy M. Miller[53], Siddharthan Chandran[54], Suvankar Pal[54], Eran Hornstein[55], Daniel J. MacGowan[56], Terry Heiman-Patterson[57], Molly G. Hammell[58], Nikolaos. A. Patsopoulos[59], Oleg Butovsky[60], Joshua Dubnau[61], Avindra Nath[62], Robert Bowser[63], Matthew Harms[64], Eleonora Aronica[3], Mary Poss[65], Jennifer Phillips-Cremins[66], John Crary[67], Nazem Atassi[68], Dale J. Lange[69], Darius J. Adams[70], Leonidas Stefanis[71,72], Marc Gotkine[25], Robert H. Baloh[73,74], Suma Babu[75], Towfique Raj[76], Sabrina Paganoni[77], Ophir Shalem[78,79], Colin Smith[80,81], Bin Zhang[82], Thomas Blanchard[35], Brent Harris[83], Iris Broce[84], Vivian Drory[26], John Ravits[85], Corey McMillan[86], Vilas Menon[87], Lani Wu[88], Steven Altschuler[88], Yossef Lerner[89], Rita Sattler[90], Kendall Van Keuren-Jensen[91], Orit Rozenblatt-Rosen[92], Kerstin Lindblad-Toh[92], Katharine Nicholson[93], Peter Gregersen[94], Jeong-Ho Lee[95], Matt Brauer[96], Shameek Biswas[97], Kimberly A Wilson[97], Sulev Koks[98], Stephen Muljo[99], Bryan J. Traynor[100], Robert Moccia[101], Seng

Cheng[101], Andrew Deubler[102], Giovanni Coppola[102], Mickey Atwal[102], Michael Cantor[102], William Salerno[102], Eli Stahl[102], Matt Anderson[102], David Frendewey[102], Daphne Koller[103], Mary Rozenman[103]

33: Center for Genomics of Neurodegenerative Disease (CGND), New York Genome Center, New York, NY
34: Department of Neurology, Lewis Katz School of Medicine, Temple University, Philadelphia, PA
35: University of Maryland Brain and Tissue Bank and NIH NeuroBioBank
36: Cedars-Sinai Department of Biomedical Sciences, Board of Governors Regenerative Medicine Institute and Brain Program, Cedars-Sinai Medical Center, and Department of Medicine, University of California, Los Angeles, CA
37: Department of Biochemistry and Molecular Biology, Penn State Institute for Personalized Medicine, The Pennsylvania State University, Hershey, PA
38: Department of Neurology, The Pennsylvania State University, Hershey, PA
39: Department of Neurology, Henry Ford Health System, Detroit, MI
40: Department of Pathology and Laboratory Medicine, Perelman School of Medicine, University of Pennsylvania, Philadelphia, PA
41: Department of Neurology, Center for Motor Neuron Biology and Disease, Institute for Genomic Medicine, Columbia University, New York, NY
42: Department of Biological Engineering, Massachusetts Institute of Technology, Cambridge, MA
43: Department of Neurology, Johns Hopkins School of Medicine, Baltimore, MD
44: Department of Neurogenetics, Academic Medical Centre, Amsterdam and Leiden University Medical Center, Leiden, The Netherlands
45: Department of Medicine, Lung Biology Center, University of California, San Francisco, CA
46: ALS Multidisciplinary Clinic, Neuromuscular Division, Department of Neurology, Harvard Medical School, and Neurological Clinical Research Institute, Massachusetts General Hospital, Boston, MA
47: Centre for Neuroscience and Trauma, Blizard Institute, Barts and The London School of Medicine and Dentistry, Queen Mary University of London, London, and Department of Neurology, Basildon University Hospital, Basildon, United Kingdom
48: Institute of Neurology, National Hospital for Neurology and Neurosurgery, University College London, London, United Kingdom
49: The Jackson Laboratory, Bar Harbor, ME
50: Department of Psychiatry & Human Behavior, Department of Biological Chemistry, School of Medicine, and Department of Neurobiology and Behavior, School of Biological Sciences, University California, Irvine, CA

51: Taube/Koret Center for Neurodegenerative Disease Research, Roddenberry Center for Stem Cell Biology and Medicine, Gladstone Institute
52: Department of Neurology & Sensory Organs, University of Thessaly, Thessaly, Greece
53: Department of Neurology, Washington University in St. Louis, St. Louis, MO
54: Centre for Clinical Brain Sciences, Anne Rowling Regenerative Neurology Clinic, Euan MacDonald Centre for Motor Neurone Disease Research, University of Edinburgh, Edinburgh, United Kingdom
55: Department of Molecular Genetics, Weizmann Institute of Science, Rehovot, Israel
56: Department of Neurology, Icahn School of Medicine at Mount Sinai, New York, NY
57: Center for Neurodegenerative Disorders, Department of Neurology, the Lewis Katz School of Medicine, Temple University, Philadelphia, PA
58: Cold Spring Harbor Laboratory, Cold Spring Harbor, NY
59: Computer Science and Systems Biology Program, Ann Romney Center for Neurological Diseases, Department of Neurology and Division of Genetics in Department of Medicine, Brigham and Women’s Hospital, Boston, MA, Harvard Medical School, Boston, MA, and Program in Medical and Population Genetics, Broad Institute, Cambridge, MA
60: Ann Romney Center for Neurologic Diseases, Brigham and Women's Hospital, Harvard Medical School, Boston, MA 7
1: Department of Anesthesiology, Stony Brook University, Stony Brook, NY
62: Section of Infections of the Nervous System, National Institute of Neurological Disorders and Stroke, NIH, Bethesda, MD
63: Department of Neurology, Barrow Neurological Institute, St. Joseph's Hospital and Medical Center, Department of Neurobiology, Barrow Neurological Institute, St. Joseph's Hospital and Medical Center, Phoenix, AZ
64: Department of Neurology, Division of Neuromuscular Medicine, Columbia University, New York, NY 14
65: Department of Biology and Veterinary and Biomedical Sciences, The Pennsylvania State University, University Park, PA
66: New York Stem Cell Foundation, Department of Bioengineering, School of Engineering and Applied Sciences, University of Pennsylvania, Philadelphia, PA
67: Department of Pathology, Fishberg Department of Neuroscience, Friedman Brain Institute, Ronald M. Loeb Center for Alzheimer's Disease, Icahn School of Medicine at Mount Sinai, New York, NY
68: Department of Neurology, Harvard Medical School, Neurological Clinical Research Institute, Massachusetts General Hospital, Boston, MA
69: Department of Neurology, Hospital for Special Surgery and Weill Cornell Medical Center, New York, NY
70: Medical Genetics, Atlantic Health System, Morristown Medical Center, Morristown, NJ, and Overlook Medical Center, Summit, NJ

71: Center of Clinical Research, Experimental Surgery and Translational Research, Biomedical Research Foundation of the Academy of Athens (BRFAA), 4 Soranou Efesiou Street, 10327, Athens, Greece
72: 1st Department of Neurology, Eginition Hospital, Medical School, National and Kapodistrian University of Athens, Athens, Greece
73: Board of Governors Regenerative Medicine Institute, Los Angeles, CA
74: Department of Neurology, Cedars-Sinai Medical Center, Los Angeles, CA
75: Neurological Clinical Research Institute, Massachusetts General Hospital, Boston, MA
76: Departments of Neuroscience, and Genetics and Genomic Sciences, Ronald M. Loeb Center for Alzheimer's disease, Icahn School of Medicine at Mount Sinai, New York, NY
77: Harvard Medical School, Department of Physical Medicine & Rehabilitation, Spaulding Rehabilitation Hospital, Boston, MA
78: Center for Cellular and Molecular Therapeutics, Children's Hospital of Philadelphia, Philadelphia, PA
79: Department of Genetics, Perelman School of Medicine, University of Pennsylvania, Philadelphia, PA
80: Centre for Clinical Brain Sciences, University of Edinburgh, Edinburgh, UK
81: Euan MacDonald Centre for Motor Neurone Disease Research, University of Edinburgh, Edinburgh, UK
82: Department of Genetics and Genomic Sciences, Icahn Institute of Data Science and Genomic Technology, Icahn School of Medicine at Mount Sinai, New York, NY
83: Department of Neuropathology, Georgetown Brain Bank, Georgetown Lombardi Comprehensive Cancer Center, Georgetown University Medical Center, Washington DC
84: Neuroradiology Section, Department of Radiology and Biomedical Imaging, University of California, San Francisco, San Francisco, CA
85: Department of Neuroscience, University of California San Diego, La Jolla, CA
86: Department of Neurology, University of Pennsylvania Perelman School of Medicine, Philadelphia, PA
87: Department of Neurology, Columbia University Medical Center, New York, NY
88: Department of Pharmaceutical Chemistry, University of California San Francisco, San Francisco, CA
89: Hadassah Hebrew University
90: Department of Translational Neuroscience, Barrow Neurological Institute, Phoenix, Arizona
91: The Translational Genomics Research Institute (TGen), Phoenix, Arizona
92: Broad Institute, Cambridge, Massachusetts
93: Massachusetts General Hospital, Boston, Massachusetts
94: Institute of Molecular Medicine, Feinstein Institutes for Medical Research, Northwell Health, Manhasset, New York
95: Korea Advanced Institute of Science and Technology (KAIST), Daejeon, South Korea

96: Maze Therapeutics
97: Bristol-Myers Squibb
98: Perron Institute for Neurological and Translational Science
99: Integrative Immunobiology Section, National Institute of Allergy and Infectious Disease, NIH
100: Neuromuscular Disease Research Section, National Institute of Aging
101: Pfizer
102: Regeneron
103: Insitro
